# Tumourigenic non-small-cell lung cancer mesenchymal circulating tumour cells: a clinical case study

**DOI:** 10.1093/annonc/mdw122

**Published:** 2016-03-24

**Authors:** C. J. Morrow, F. Trapani, R. L. Metcalf, G. Bertolini, C. L. Hodgkinson, G. Khandelwal, P. Kelly, M. Galvin, L. Carter, K. L. Simpson, S. Williamson, C. Wirth, N. Simms, L. Frankliln, K. K. Frese, D. G. Rothwell, D. Nonaka, C. J. Miller, G. Brady, F. H. Blackhall, C. Dive

**Affiliations:** 1 Clinical and Experimental Pharmacology Group, University of Manchester, Manchester, UK; 2 Tumour Genomics Unit, Department of Experimental Oncology and Molecular Medicine, Fondazione IRCCS Istituto Nazionale dei Tumori, Milan, Italy; 3 RNA Biology Group, University of Manchester, Manchester; 4 Computational Biology Support Team, Cancer Research UK Manchester Institute, University of Manchester, Manchester; 5 The Christie NHS Foundation Trust,Manchester; 6 Institute of Cancer Sciences, University of Manchester, Manchester; 7 Cancer Research UK Lung Cancer Centre of Excellence, Manchester, UK

**Keywords:** non-small-cell lung cancer, circulating tumour cells, patient-derived circulating tumour cells explants (CDX), preclinical therapeutics, epithelial to mesenchymal transition (EMT)

## Abstract

**Background:**

Over the past decade, numerous reports describe the generation and increasing utility of non-small-cell lung cancer (NSCLC) patient-derived xenografts (PDX) from tissue biopsies. While PDX have proven useful for genetic profiling and preclinical drug testing, the requirement of a tissue biopsy limits the available patient population, particularly those with advanced oligometastatic disease. Conversely, ‘liquid biopsies’ such as circulating tumour cells (CTCs) are minimally invasive and easier to obtain. Here, we present a clinical case study of a NSCLC patient with advanced metastatic disease, a never smoker whose primary tumour was EGFR and ALK wild-type. We demonstrate for the first time, tumorigenicity of their CTCs to generate a patient CTC-derived eXplant (CDX).

**Patients and methods:**

CTCs were enriched at diagnosis and again 2 months later during disease progression from 10 ml blood from a 48-year-old NSCLC patient and implanted into immunocompromised mice. Resultant tumours were morphologically, immunohistochemically, and genetically compared with the donor patient's diagnostic specimen. Mice were treated with cisplatin and pemetrexed to assess preclinical efficacy of the chemotherapy regimen given to the donor patient.

**Results:**

The NSCLC CDX expressed lung lineage markers TTF1 and CK7 and was unresponsive to cisplatin and pemetrexed. Examination of blood samples matched to that used for CDX generation revealed absence of CTCs using the CellSearch EpCAM-dependent platform, whereas size-based CTC enrichment revealed abundant heterogeneous CTCs of which ∼80% were mesenchymal marker vimentin positive. Molecular analysis of the CDX, mesenchymal and epithelial CTCs revealed a common somatic mutation confirming tumour origin and showed CDX RNA and protein profiles consistent with the predominantly mesenchymal phenotype.

**Conclusions:**

This study shows that the absence of NSCLC CTCs detected by CellSearch (EpCAM^+^) does not preclude CDX generation, highlighting epithelial to mesenchymal transition and the functional importance of mesenchymal CTCs in dissemination of this disease.

## Abstract (precis)

An explant model derived from EpCam negative mesenchymal non-small-cell lung (NSCLC) cancer circulating tumour cells (a ‘liquid biopsy’) recapitulates the histology of the donor patient's diagnostic specimen and chemoresistance to cisplatin and pemetrexed. This proof-of-principal landmark model opens a new avenue for study of advanced NSCLC biology when tissue biopsies unavailable.

## introduction

Interest in circulating tumour cells (CTCs) as ‘liquid biopsies’ has escalated as technical hurdles are overcome [1]. Recently, we and others increased the scope of CTC research, demonstrating for small-cell lung cancer (SCLC) and breast cancer, that CTCs are tumorigenic in immunocompromised mice [2–4], creating novel CTC-derived eXplant models (CDX). Patients whose blood samples gave rise to CDX had >400 CTCs/7.5 ml blood assessed using the epithelial marker-dependent (EpCAM^+^/pan-cytokeratin^+^) CellSearch platform, yet many highly metastatic cancers have considerably lower epithelial CTC counts. Only 32% of stage IV non-small-cell lung cancer (NSCLC) patients had ≥2 CellSearch CTCs/7.5 ml blood [5] yet when CTC number was defined as CD45^−^ cells unable to pass through 8 µm filter pores, this percentage rose to 83% [6]. Filtered NSCLC CTCs also revealed high prevalence of vimentin expression [7, 8], consistent with mesenchymal phenotype. The following case study supports our hypothesis that advanced NSCLC patients' blood (even in the absence of CellSearch CTCs) contains tumorigenic mesenchymal CTCs of functional importance for metastasis.

## materials and methods

### patient recruitment and blood collection

A patient with histologically confirmed chemotherapy naïve NSCLC who was referred to The Christie Hospital NHS Trust provided written informed consent which specified their samples could be used for *in vivo* studies and genetic analysis in accordance with UK regulatory requirements. The study was prospectively approved by the NHS NorthWest 9 Research Ethical Committee.

Blood was drawn at baseline, before administration of chemotherapy, and again after completion of brain radiotherapy. Three 10 ml blood samples were taken at each time point; two into EDTA vacutainers (Becton Dickinson) for CTC enrichment before implantation into immunocompromised mice and for ISET filtration of CTCs, and one into a CellSave vacutainer (Jansen Diagnostics), for CellSearch CTC enumeration.

### CTC enrichment before implantation into mice

Within 30 min of blood draw, a 10 ml sample of blood in an EDTA tube was processed using the RosetteSep Human Circulating Epithelial Tumour Cell Cocktail (Stem Cell Technology) according to the manufacturer's instructions and as previously described [2].

### growth of CDX in immunocompromised mice

All procedures were carried out as previously described [2] in accordance with Home Office Regulations (UK) and the UK Coordinating Committee on Cancer Research guidelines and by locally approved protocols (Home Office Project licence no. 40-3306). In some instances, CDX were passaged following disaggregation using a human tumour dissociation kit (Miltenyi Biotech) following the manufacturer's instructions. Dead cells were removed from the disaggregated tumour with a dead cell removal kit (Miltenyi Biotech) following the manufacturer's instructions. Murine cells were removed by mixing 20 µl anti-mouse IgG2a+b microbeads (Miltenyi Biotech), 10 µl anti-mouse MHC Class I antibody (eBioscience), and 500 µl binding buffer (Miltenyi Biotech), incubating at 4°C for 30 min, mixing with disaggregated tumour cells and incubating at room temperature for 15 min. Cell–bead mixture was then applied to an LS column (Miltenyi Biotech) in a MidiMACS separator (Miltenyi Biotech), the flow through collected, the column washed with 4 × 3 ml binding buffer, and the flow through and wash containing the human cells combined. Disaggregated cells were collected by centrifugation, resuspended in 10% DMSO in fetal bovine serum (Biowest) and stored at −80°C or in liquid nitrogen before re-implantation.

### cisplatin/pemetrexed treatment of CDX

Twenty-four 10- to 12-week-old female CB-17/lcrHsd-*Prkdc^scid^Lyst^bg-J^* (SCID-*bg*) mice (Harlan) were implanted with 100 000 disaggregated CDX cells from a passage 3 CDX tumour in 100 μl of 1:1 RPMI (Life Technologies):matrigel. As the first 20 tumours reached 200–250 mm^3^, the mice were randomized by sequential assignment to cisplatin/pemetrexed or vehicle treatment groups. Animals were treated by i.p. injection with 200 mg/kg pemetrexed (Alimta—Eli Lilly), dissolved to 20 mg/ml with 0.9% saline solution, on day 1 followed 1 h later with 5 mg/kg cisplatin (Hospira), supplied as a 1 mg/ml solution in 0.9% saline solution, or corresponding vehicle only. Tumour volume was monitored blinded to treatment group three times a week until tumour reached 1000 mm^3^.

### immunohistochemistry

TTF-1 and CK7 IHC was carried out on formalin-fixed, paraffin-embedded tissue 4 μm sections using anti-TTF-1 (mouse, 8GTG3/1, 1:200, Dako, Carpinteria, CA, USA) and anti-CK7 (mouse, OV-TL 12/30, 1:1000, Dako). Antibody incubation and detection were carried out at 37°C on a Menarini intelliPATH FLX (A. Menarini Diagnostics, UK) using Menarini's reagent buffer and detection kits unless otherwise noted. Antigen retrieval was carried out in a pressure cooker in citrate buffer pH6 for 2 min at 123°C. Pan-CK, CD44 and vimentin IHC was carried out on formalin-fixed, paraffin-embedded tissue 4 μm sections using anti pan-cytokeratin antibody (mouse, M3515, 1:60, ER1 20 min Dako), anti CD44 (mouse, DF1485, 1:100, ER2 10 min, Dako) and anti-Vimentin (mouse, V9, CC1 32 min, Roche). Pan-CK and CD44 staining was carried out on the LEICA Bond Max Platform and Vimentin staining on the Ventana Discovery Ultra platform (Roche). Appropriate positive, negative and isotype controls were included with the study sections (not shown). Digital images of whole-tissue sections acquired using a Leica SCN400 histology scanner (Leica Microsystems).

### CellSearch CTC enumeration

Blood drawn into a CellSave tube was analysed for the presence of CTCs using the CellSearch platform and CTC kit (Jansen Diagnostics) according to the manufacturer's instructions and as previously described [7]. A cell was defined as a CTC if it expressed EpCAM and CK, was >4 µm in diameter, had an intact DAPI-stained nuclei, and did not express CD45.

### ISET CTC enrichment, immunofluorescence staining, and epithelial/mesenchymal phenotype scoring

Ten millilitres of blood drawn into an EDTA tube were stored at 4°C and processed by ISET filtration (Rarecells) within 4 h of collection according to the manufacturer's instructions and as previously described [6] and stored at −20°C. ISET-filtered CTCs were stained by multi-parameter immunofluorescence using antibodies against CD45 (leukocyte marker), pan-cytokeratins (epithelial cell marker), vimentin (mesenchymal cell marker), and VE-cadherin (CD144, endothelial cell marker). Filters were rehydrated (1 min in PBS + 0.02% Tween-20), permeabilized (0.2% Triton X-100 for 10 min), and washed for 1 min in PBS + 0.02% Tween-20. Antibody incubations were as follows: 10% goat serum block (Dako X0907) for 30 min; a cocktail (80 µl) of two primary antibodies, unconjugated anti-CD45 (1:400 of 0.4 mg/ml, Rabbit, Abcam AB10559), and unconjugated anti-VE-cadherin (1:200 of 0.5 mg/ml, Mouse, eBioscience 14-1449-82), for 1 h; a cocktail (80 µl) of two fluorescent secondary antibodies, Goat anti-mouse AF555 (1:500 of 2 mg/ml Invitrogen A21422) and Goat anti-rabbit DY549 (1:500 of 1 mg/ml Thermo Scientific35 558) for 1 h; a cocktail (80 µl) of two conjugated primary antibodies, anti-vimentin 647 (1:50 of 0.05 mg/ml Santa Cruz sc-6260) and anti-CK FITC (1:100 of 3 mg/ml, Sigma F3418, clone C11 against CKs 4, 5, 6, 8, 10, 13, and 18) for 1 h; and 80 µl of DAPI (1:10 000 of 5 mg/ml, Invitrogen D3571) for 5 min. The filters were washed twice in PBST between incubations then twice in double distilled water before mounting with ProLong Gold anti-fade (Invitrogen P36934). Slides were left overnight at room temperature to dry and imaged with a MIRAX automated slide scanner (3DHistech, Budapest, Hungary). Images were reviewed with 3DHistech panoramic viewing software (Version 1.15.1.10). This assay detects CD45 and VE-cadherin positive cells (leucocytes and endothelial cells) with the same fluorescence emission (both labelled with fluorophores with red spectral emission). Therefore, tumour cells in this assay were at the outset of the study defined as having neither marker (i.e. CTCs are defined as cytokeratin and/or vimentin positive and CD45 and VE-cadherin negative). Expression of vimentin and/or cytokeratins on filtered cells was evaluated by analysts with extensive experience in CTC research.

### whole-exome sequencing

The CDX tumours were disaggregated using a sterile scalpel and gDNA isolated using the QiaAmp DNA Mini kit (Qiagen). DNA libraries were then generated from 750 ng gDNA in the NEBNext Ultra DNA Library kit (NEB) and enriched for exome-specific sequences using the SureSelect Human All Exon V5 kit (Agilent) following the manufacturer's instructions. Resulting libraries were then sequenced on an Illumina HiSeq2500 instrument using the TruSeq PE Cluster Kit V3 and TruSeq SBSv3 chemistry. Bioinformatic analysis was carried out as previously described [2].

### single-cell laser capture microdissection

A spot of an ISET filter stained for multiple markers as described above was washed with water, dried and mounted on a membrane-free LCM metal-framed slide. Cells were visualized on a Leica LMD6000 LCM microscope (Leica Microsystems) at 60× magnification and epithelial, mesenchymal, and mixed phenotype CTCs and leucocytes/endothelial cells were identified. Cells were dissected under direct immunofluorescent microscopic visualization and dropped directly into the lid of a 0.2 ml collection tube. Twelve single CTCs, four pools of two out of three CTCs eight single leucocytes/endothelial cells, and four pools of three out of four leucocytes/endothelial cells were micro-dissected and used for subsequent downstream analysis. The cells selected via fluorescence attributed to CD45 or VE-Cadherin (CD44) were small cells and more likely to be leucocytes than endothelial cells.

### Sanger sequencing

Captured cells were centrifuged at 15 000*g* for 10 min. DNA was extracted and amplified with a Picoplex Whole Genome Amplification kit (Rubicon) according to the manufacturer's instructions. Products of DNA amplification were checked on an agarose gel 1.5%, purified using the High Pure PCR Kit (Roche) and quantified using Qubit fluorometer (Life Technologies). Sanger sequencing assays to detect 18 somatic mutations were tested on the amplified DNA, but due to technical inefficiencies, only the assay to detect a G340A mutation in PACRG proving fit for purpose. In brief, a portion of the PACRG gene containing the mutated locus was amplified using primers PACRG-F1 (5′-GCCCGAATGCTGTTTTCACA) and PACRG-R1 (5′-GGTTGTCTGGCCTCCTAAGT) with PCR cycling conditions of 98°C for 20 s, 59°C for 30 s, 72°C for 30 s for 33 cycles using TaKaRa Ex Taq HS DNA Polymerase (TaKaRa Bio). The resulting PCR product was then purified using QIAquick PCR Purification Kit (Qiagen) and directly sequenced.

### RNAseq

CDX-derived tumour tissue fragments were collected from autopsied animals into RNAlater (Sigma) and stored at −80°C. Tumours were manually disaggregated using a sterile scalpel, before undergoing RNA extraction by miRNeasy mini kit (Qiagen) following the manufacturer's instructions. Next-generation sequencing libraries were generated by SureSelect Poly A kit (Aligent), before undergoing 150 bp Paired End sequencing on a NextSeq 500 sequencer (Illumina). The RNASeq data were aligned to Human GRCh38 and Mouse GRCm38 assembly using Mapsplice (Version 2.1.6). Reads aligning to the mouse genome were removed from the set of human-aligned reads. Filtered reads were then used to generate counts data using Rsubread (Version 1.16.1) with Ensembl version 77 GTF file. Counts were converted into Reads Per Kilobase per Million mapped (RPKM), which were then used for expression level quantification of the genes.

## results

A 48-year-old male was recruited through an ongoing, ethically approved translational lung cancer research programme. Diagnostic computer tomography identified a T1aN2M1b stage tumour (TNM 7th edition [9]) with a 2 cm right lung primary and metastases to brain, bone, kidney, mediastinal, and para-aortic lymph nodes. His diagnostic biopsy (a para-aortic lymph node) contained sheets of polygonal poorly differentiated adenocarcinoma cells that were positive for lung markers, thyroid transcription factor (TTF1), and cytokeratin7 (CK7) and negative for p40 (squamous marker), CD56, chromogranin A, synaptophysin (neuroendocrine markers), and CK20 (gastrointestinal marker). Molecular testing demonstrated wild-type EGFR and was negative for ALK gene rearrangement. The patient commenced on chemotherapy with cisplatin and pemetrexed but discontinued after one cycle with deterioration of his general condition due to brain metastases. Despite administration of whole-brain radiotherapy (20 Gy, 5 fractions), the patient continued to deteriorate with progressive neurological symptoms and died 2 months following initial diagnosis. Three blood samples were drawn before chemotherapy (baseline) and after brain radiotherapy. After blood cell depletion from one sample, the CTC-enriched fraction was implanted into an immunocompromised mouse within 2 h of blood draw to attempt CDX generation. The other samples were processed for CTC analysis using CellSearch and ISET filtration.

**Figure 1.**
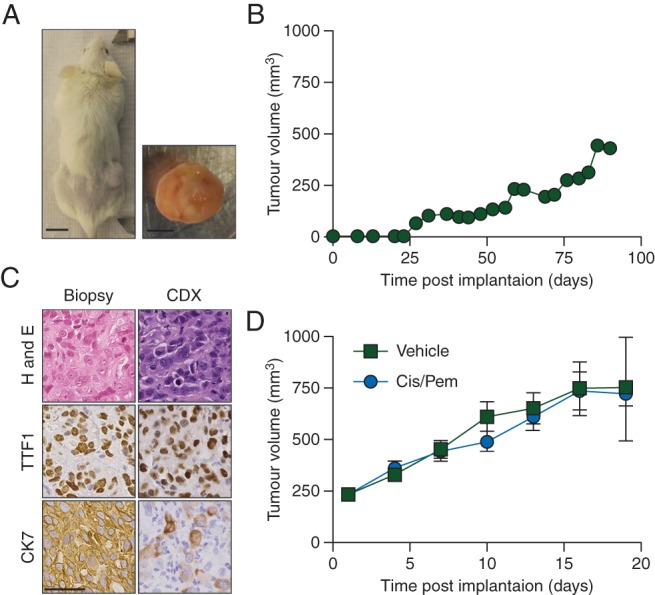
NSCLC CDX growth, histology and response to therapy. (A) Mouse bearing a passage 2 CDX derived from CTCs enriched from an NSCLC patient (scale bar, 1 cm) and the resected tumour (scale bar, 5 mm). (B) Size of CDX tumour shown in A versus time post-implantation. (C) Patient biopsy and CDX shown in A stained for H&E, TTF1, and CK7 (scale bar, 50 µm). (D) Twenty-four SCID-*bg* mice were implanted with 100 000 disaggregated cells from a passage 4 CDX. When tumours reached ∼200 mm^3^, they were randomly assigned to a vehicle control group (*n* = 10) or 5 mg/kg cisplatin bolus, 200 mg/kg pemetrexed bolus-treated group (*n* = 10). Tumour volume was monitored two to three times a week.

No CDX was derived at baseline, but post-radiotherapy CTCs gave rise to a palpable tumour 95 days after implantation; CDX fragments implanted into additional mice resulted in palpable tumours ∼30 days later (Figure 1A and B). The CDX resembled a poorly differentiated lung adenocarcinoma, comprising diffuse sheets of large polygonal cells with abundant eosinophilic cytoplasm, vesicular chromatin, and enlarged nucleoli (Figure 1C). Both biopsy and CDX expressed TTF1. CK7 was expressed in ∼10% versus 100% CDX cells versus biopsy, suggesting a less epithelial CDX phenotype (Figure 1C). The CDX, when treated with cisplatin and pemetrexed (the same chemotherapy doublet as the patient), was resistant to treatment (no impact on tumour growth) suggesting that had progressive neurological symptoms not prevented the patient's continuation of chemotherapy, treatment may still not have improved the outcome (Figure 1D).

**Figure 2.**
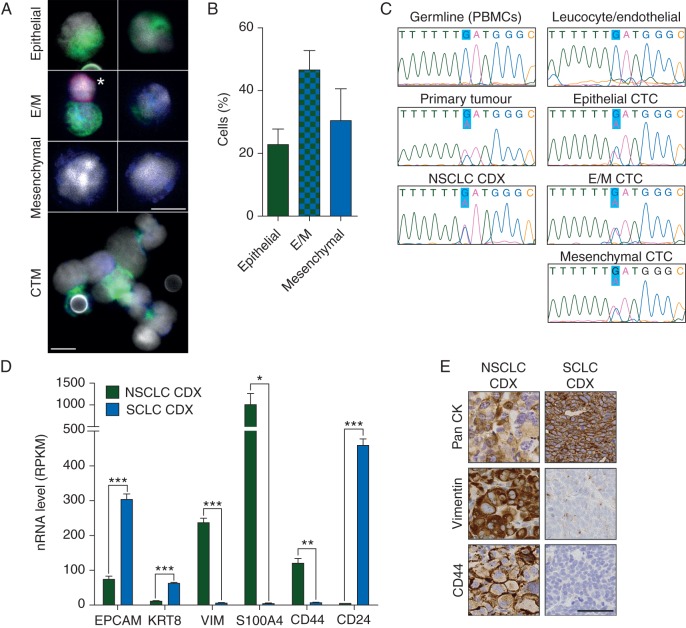
Epithelial/mesenchymal nature of patient CTCs and CDX. CTCs were enriched via ISET filtration from a parallel blood sample to that which generated the CDX. The ISET filter was stained for pan-CK (green), vimentin (blue), CD45 and CD144 (pink), and DAPI (white). (A) Representative images and (B) quantitation of cells classed as epithelial, mixed epithelial/mesenchymal (E/M), and mesenchymal. Also shown is a circulating tumour microemboli (CTM) containing cells of all three phenotypes. *Leucocyte or endothelial cell; scale bar, 10 µm. Quantitation carried out by two independent scorers on two ISET spots, each counting >150 CTCs per spot. Error bars, SEM. (C) Single cells from the ISET filter shown in A classed as a leucocyte/endothelial cell, epithelial CTC, mixed E/M CTC, or a mesenchymal CTC were captured by laser capture microdissection. The PACRG locus, shown by WES to be mutated in the CDX, was Sanger sequenced in DNA extracted from the captured CTCs and green fluorescent cells (leucocytes or endothelial cells), from the patients PBMCs (germline sample), from their primary tumour biopsy and from the passage 2 CDX shown in Figure 1A–C. Highlighted base represents the mutated base. (D) RNAseq was carried out on passage 2 NSCLC CDX tumours and passage 3 CDX from two SCLC CDX models. Expression of EPCAM, KRT8, VIM, S100A4, CD44, and CD24 is displayed as RPKM values. Data represent mean of three independent tumours ± SEM. *P < 0.05; ***P < 0.001 according to two-tailed unpaired *t*-test. (E) Tumours analysed in D were stained for expression of pan-CK, vimentin, and CD44 (scale bar, 50 µm).

CellSearch analysis detected four EpCAM^+^/CK^+^ CTCs/7.5 ml blood at baseline and zero CTCs post-radiotherapy. Multi-parameter immunofluorescence on the post-radiotherapy filtered blood sample blood revealed >150 CTCs (CD45^−^/CD144^−^) cells/ml comprising epithelial CK^+^/vimentin^−^ (23%), mesenchymal CK^−^/vimentin^+^ (30%), and mixed phenotype CK^+^/vimentin^+^ (47%) CTCs; circulating tumour microemboli containing cells of all three phenotypes were also detected (Figure 2A and B). CDX whole-exome sequencing (supplementary Table 1, available at *Annals of Oncology* online) revealed somatic mutations in 247 genes, including TP53 and KEAP1, commonly mutated in NSCLC. To confirm tumour origin of filtered CTCs, we sought common mutations in tumour biopsy, CDX, and cells designated as CTCs (retrieved by laser capture microdissection for genomic DNA extraction and amplification) not present in leucocytes/endothelial cells. Confirmation of CTC tumour origin (by Sanger sequencing) was demonstrated by the absence of a G340A mutation in PACRG in germline DNA present in primary tumour, CDX, and single epithelial, mesenchymal, and mixed phenotype CTCs (Figure 2C). Expression of genes associated with epithelial or mesenchymal phenotypes [10, 11] was evaluated after RNAseq of passage 3 CDX (Figure 2D, supplementary Table 2, available at *Annals of Oncology* online). Consistent with a mesenchymal phenotype, the NSCLC CDX expressed low levels of epithelial genes EPCAM and KRT8, high levels of mesenchymal genes vimentin and S100A4 [11], and a high ratio of CD44:CD24 [10]. The NSCLC CDX expressed low protein levels of pan-CK and high levels of vimentin and CD44 (Figure 2E). These RNA and protein profiles contrasted with a SCLC CDX from a donor patient with 458 EpCAM^+^/CK^+^ CTCs/7.5 ml blood [2] which displayed a strong epithelial phenotype.

## discussion

We report for the first time, NSCLC circulating cells with tumour initiating potential and CDX generation from a patient without detectable EpCAM^+^/CK^+^ CTCs [2, 3]. These data highlight the potential for CDX models from metastatic cancers with low prevalence of CellSearch CTCs (e.g. pancreatic cancer) and where biopsy material is often scarce, resulting in insufficient material for comprehensive molecular profiling. Our data also denote the importance of non-epitope-dependent technologies for CTC enrichment [12, 13]. While we do not formally distinguish dynamic EMT from epithelial and mesenchymal cell cooperation during haematological dissemination, these data do highlight a functional importance of mesenchymal CTCs and need for treatment strategies that target them (e.g. AXL inhibitors) [14]. The persistent, predominant mesenchymal phenotype of the established CDX may also suggest that mesenchymal to epithelial transition (MET) [11] may not always reverse after NSCLC cell dissemination.

In this case, the patient died to his disease 1 day after the blood draw that generated the CDX. This exemplifies how CDX can represent end of life disease models and the pressing need to better understand progressive disease biology and therapeutic options for advanced patients. The CDX approach contrasts with the majority of patient-derived xenografts (PDX) generated from resected tissue during surgery with curative intent [15]. CDX can be generated from patients with little deviation from routine practice and this study demonstrates the potential for this approach to be applied to cancers with few CellSearch detectable CTCs. It remains to be determined how frequently CDX could be generated from NSCLC patients with no/few CellSearch detectable CTCs. At the time of submission, we had implanted 34 samples enriched for CTC from NSCLC patients into mice with no further CDX yet detectable. However, the time from CTC implant to CDX detection can be over 6 months, so this dataset needs to mature before conclusions regarding CDX ‘take rate’ can be drawn. This time frame precludes the use of NSCLC CDX as ‘avatars’ in co-clinical trials to direct patient treatment. However, if CDX can be routinely generated from NSCLC patients, they have the potential to provide paired models from the same patient at baseline and at progression to study drug resistance mechanisms. We suggest that CDX would represent optimal preclinical models to test novel therapeutic regimens before commencing early clinical trials, as they can be generated from the cohort of patients better representing those at trial entry.

## funding

This research was supported by the British Lung Foundation (R914-6); Cancer Research UK (C5759/A12328); the Manchester CRUK Experimental Cancer Medicine Centre (C1467/A15578); the Manchester Cancer Research Centre (A12197); CRUK Lung Cancer Centre of Excellence (C5759/A20465); European Union CHEMORES FP6 (LSHG-CT-2007-037665); and travel grants to Guilia Bertonlini from Associazione Italiana Ricerca Cancro, Accademia Nazionale dei Lincei, and Progetto Professionalità_Fondazione Banca del Monte della Lombardia.

## disclosure

The authors have declared no conflicts of interest.

## Supplementary Material

Supplementary Data

## References

[1] Krebs MG, Metcalf RL, Carter L et al Molecular analysis of circulating tumour cells-biology and biomarkers. Nat Rev Clin Oncol 2014; 11: 129–144. 24445517 10.1038/nrclinonc.2013.253

[2] Hodgkinson CL, Morrow CJ, Li Y et al Tumorigenicity and genetic profiling of circulating tumor cells in small-cell lung cancer. Nat Med 2014; 20: 897–903. 24880617 10.1038/nm.3600

[3] Baccelli I, Schneeweiss A, Riethdorf S et al Identification of a population of blood circulating tumor cells from breast cancer patients that initiates metastasis in a xenograft assay. Nat Biotechnol 2013; 31: 539–544. 23609047 10.1038/nbt.2576

[4] Yu M, Bardia A, Aceto N et al Cancer therapy. Ex vivo culture of circulating breast tumor cells for individualized testing of drug susceptibility. Science 2014; 345: 216–220. 25013076 10.1126/science.1253533 PMC4358808

[5] Krebs MG, Sloane R, Priest L et al Evaluation and prognostic significance of circulating tumor cells in patients with non-small-cell lung cancer. J Clin Oncol 2011; 29: 1556–1563. 21422424 10.1200/JCO.2010.28.7045

[6] Krebs MG, Hou JM, Sloane R et al Analysis of circulating tumor cells in patients with non-small cell lung cancer using epithelial marker-dependent and -independent approaches. J Thorac Oncol 2012; 7: 306–315. 22173704 10.1097/JTO.0b013e31823c5c16

[7] Hou JM, Krebs M, Ward T et al Circulating tumor cells as a window on metastasis biology in lung cancer. Am J Pathol 2011; 178: 989–996. 21356352 10.1016/j.ajpath.2010.12.003 PMC3069884

[8] Lecharpentier A, Vielh P, Perez-Moreno P et al Detection of circulating tumour cells with a hybrid (epithelial/mesenchymal) phenotype in patients with metastatic non-small cell lung cancer. Br J Cancer 2011; 105: 1338–1341. 21970878 10.1038/bjc.2011.405 PMC3241564

[9] Goldstraw P, Crowley J, Chansky K et al The IASLC Lung Cancer Staging Project: proposals for the revision of the TNM stage groupings in the forthcoming (seventh) edition of the TNM Classification of malignant tumours. J Thorac Oncol 2007; 2: 706–714. 17762336 10.1097/JTO.0b013e31812f3c1a

[10] Mani SA, Guo W, Liao MJ et al The epithelial–mesenchymal transition generates cells with properties of stem cells. Cell 2008; 133: 704–715. 18485877 10.1016/j.cell.2008.03.027 PMC2728032

[11] Thiery JP, Acloque H, Huang RY, Nieto MA Epithelial–mesenchymal transitions in development and disease. Cell 2009; 139: 871–890. 19945376 10.1016/j.cell.2009.11.007

[12] de Bono JS, Scher HI, Montgomery RB et al Circulating tumor cells predict survival benefit from treatment in metastatic castration-resistant prostate cancer. Clin Cancer Res 2008; 14: 6302–6309. 18829513 10.1158/1078-0432.CCR-08-0872

[13] Khoja L, Backen A, Sloane R et al A pilot study to explore circulating tumour cells in pancreatic cancer as a novel biomarker. Br J Cancer 2012; 106: 508–516. 22187035 10.1038/bjc.2011.545 PMC3273340

[14] Byers LA, Diao L, Wang J et al An epithelial–mesenchymal transition gene signature predicts resistance to EGFR and PI3K inhibitors and identifies Axl as a therapeutic target for overcoming EGFR inhibitor resistance. Clin Cancer Res 2013; 19: 279–290. 23091115 10.1158/1078-0432.CCR-12-1558 PMC3567921

[15] Hidalgo M, Amant F, Biankin AV et al Patient-derived xenograft models: an emerging platform for translational cancer research. Cancer Discov 2014; 4: 998–1013. 25185190 10.1158/2159-8290.CD-14-0001 PMC4167608

