# Supplementary material for: Tumourigenic non-small-cell lung cancer mesenchymal circulating tumour cells: a clinical case study
Source: Ann Oncol. 2016 Mar 24;27(6):1155–60. doi: 10.1093/annonc/mdw122 (PMC4880063; doi:10.1093/annonc/mdw122)
Supplement: Supplementary Data [file supp_27_6_1155__index.html]

Tumourigenic Non-Small Cell Lung Cancer Mesenchymal Circulating Tumour Cells - A Clinical Case Study — Tumourigenic non-small-cell lung cancer mesenchymal circulating tumour cells: a clinical case study — Tumourigenic non-small-cell lung cancer mesenchymal circulating tumour cells: a clinical case study — Supplementary Data 

# Tumourigenic non-small-cell lung cancer mesenchymal circulating tumour cells: a clinical case study

## Supplementary Data

Supplementary Data

- Supplementary Table 1 - xlsx file
- Supplementary Table 2 - xlsx file
